# Red blood cell membrane-enveloped O_2_ self-supplementing biomimetic nanoparticles for tumor imaging-guided enhanced sonodynamic therapy

**DOI:** 10.7150/thno.37930

**Published:** 2020-01-01

**Authors:** Cheng Li, Xiao-Quan Yang, Jie An, Kai Cheng, Xiao-Lin Hou, Xiao-Shuai Zhang, Yong-Guo Hu, Bo Liu, Yuan-Di Zhao

**Affiliations:** 1 Britton Chance Center for Biomedical Photonics at Wuhan National Laboratory for Optoelectronics-Hubei Bioinformatics & Molecular Imaging Key Laboratory, Department of Biomedical Engineering, College of Life Science and Technology, Huazhong University of Science and Technology, Wuhan 430074, Hubei, P. R. China;; 2 Beijing Advanced Innovation Center for Biomedical Engineering, Beijing Advanced Innovation Center for Big Data-Based Precision Medicine, School of Medicine, Beihang University, Beijing, 100083, P. R. China;; 3 Key Laboratory of Biomedical Photonics (HUST), Ministry of Education, Huazhong University of Science and Technology, Wuhan 430074, Hubei, P. R. China.

**Keywords:** tumor hypoxia, sonodynamic therapy, red blood cells, nanomedicine, cancer therapy.

## Abstract

Non-invasive sonodynamic therapy (SDT) was developed because of its advantages of high penetration depth and low side effects; however, tumor hypoxia greatly restricts its therapeutic effect. In this study, we aimed to develop ideal O_2_ self-supplementing nanoparticles for imaging-guided enhanced sonodynamic therapy of tumors with the adept coalescence of biology with nanotechnology.

**Methods:** Based on the natural enzyme system of red blood cells (RBC), biomimetic nanoparticles (QD@P)Rs were fabricated by encapsulating Ag_2_S quantum dots (QD) in RBC vesicle membranes. The anti-tumor drug PEITC was employed to increase the intracellular H_2_O_2_ concentration in tumor cells.

**Results:*** In vitro* and *in vivo* experiments demonstrated excellent biocompatibility and prolonged blood circulation of (QD@P)Rs. Following oral administration of PEITC in mice to improve the H_2_O_2_ concentration, the enzyme in the nanoprobe catalyzed endogenous H_2_O_2_ to increase O_2_ content and effectively alleviate tumor hypoxia. Triggered by ultrasound under the guidance of fluorescence imaging, (QD@P)Rs generated reactive oxygen species (ROS) to induce tumor cell death, and the increased content of O_2_ significantly enhanced the effect of SDT.

**Conclusion:** Ag_2_S QDs were used, for the first time, as a sonosensitizer in the SDT field. In this study, we integrated the advantages of the natural enzyme system and SDT to develop a novel approach for effective non-invasive treatment of cancer.

## Introduction

Advances in cancer diagnosis and treatment technology play an important role in improving patient survival. There is an urgent need to develop non-invasive or minimally invasive therapeutic modalities to avoid the severe side effects of traditional approaches, such as surgical resection, radiotherapy, and chemotherapy 1-3. Photodynamic therapy (PDT), which generates reactive oxygen species (ROS) by employing light to activate photosensitizers, has been widely used in clinical diagnosis and treatment of surface tumors as a non-invasive tumor therapy 4-6. However, due to the limited tissue-penetrating depth of light, PDT is not satisfactory for the treatment of deep-seated tumors 7, 8. Ultrasound (US) as a mechanical wave has been extensively explored in clinical diagnosis and therapy, such as US imaging and high-intensity focused US 9, 10. Besides, low-intensity US is also capable of triggering some substances, known as sonosensitizers, and generating ROS for cancer therapy termed sonodynamic therapy (SDT) 11-14. Compared with PDT, SDT has the advantages of deep penetration, low cost, and high safety by using US as a stimulation source, and is, therefore, a promising non-invasive treatment for deep-seated tumors. At present, the widely reported sonosensitizers are mainly organic molecules with specific sonosensitization capability 15, 16, such as hematoporphyrin, photofrin, methylene blue, and chlorin. However, the low bioavailability and unstable chemical/biological properties of these sensitive agents lead to unsatisfactory SDT therapeutic effect and phototoxicity in sunlight. Therefore, it is highly desirable to develop new sonosensitizers with stable performance and low phototoxicity. In recent years, some nanoparticles were developed with a satisfactory sonochemical performance that could compensate for the deficiencies of organic sonosensitizers for SDT, including TiO_2_ 17, black phosphorus 18, and porphyrin-based metal organic framework 19.

The process of SDT utilizes O_2_ as one of the main sources of ROS. However, due to aberrant cell proliferation, abnormal vasculature, and dysfunction of the lymphatic system, tumor hypoxia is considered to be one of the hostile features of solid tumors, which severely restricts the therapeutic effect of SDT. Simultaneously, the rapid consumption of oxygen at the tumor site during SDT can also cause a domino effect, and the locally aggravated hypoxia further inhibit SDT resulting in compromised therapeutic effect and poor prognosis 20. Recent studies have shown that hypoxia can also lead to tumor resistance to chemotherapy and radiotherapy 21. Therefore, to combat hypoxia-mediated resistance and achieve a better therapeutic effect, exploring tumor-targeted O_2_ delivery and intratumoral O_2_ production strategies to upregulate the O_2_ content in the tumor have become central to SDT as well as PDT. Zhang and co-workers employed MnO_2_ nanosheet to decompose endogenous H_2_O_2_ in tumor cells and generate O_2_, thus overcoming hypoxia and improving PDT treatment effect 22. Liu et al. developed a strategy to modulate tumor hypoxia by taking advantage of the high oxygen dissolving ability of perfluorocarbon, which promoted tumor oxygenation efficiency by transporting oxygen from lungs to the tumor site to overcome the hypoxia-associated resistance in PDT and radiotherapy 20. In another study, targeted transportation of a catalase-loaded nanocarrier to the tumor site catalyzing the high content of H_2_O_2_ in cells to increase the supply of oxygen was also an effective way to improve the therapeutic effect 23.

Artificial materials often trigger an immune response from the body's immune defense system and, therefore necessitate stringent requirements during application *in vivo*. Inspired by the long circulation, good biocompatibility, and low immunogenicity of biological membranes 24, 25, several studies employed membranes of red blood cells (RBCs) 26, 27, cancer cells 28, platelets 29, 30 and stem cells 31 to construct biomimetic nanocarriers. For example, Zhang and colleagues applied the plasma membrane of human platelets to enclose polymeric nanoparticles with a right-side-out unilamellar membrane coating functionalized with immunomodulatory and adhesion antigens associated with platelets 32. Liu et al. designed a fusion of platelet and leukocyte (WBC) membranes to wrap magnetic beads. These hybrid membrane-coated immunomagnetic beads inherited enhanced cancer cell-binding ability from PLTs and reduced homologous WBC interaction from WBCs and were used for highly efficient and specific isolation of circulating tumor cells 33.

RBC membranes were used by Zhang and co-workers to coat polymeric nanoparticles and develop a toxin nanosponge that could absorb membrane-damaging toxins and divert them away from their cellular targets. The nanosponge presented a detoxification treatment that could potentially treat a variety of injuries and diseases caused by pore-forming toxins 34. Zhao et al. prepared RBC membrane-coated Fe_3_O_4_ nanoparticles reliant on CD47 marker on the RBC surface to escape immune clearance through interactions with the signal regulatory protein-alpha (SIRP-α) receptor 35. Also, RBCs, as the main oxygen carrier in mammals, are needed for the transportation and regulation of oxygen. RBCs are lacking the nucleus and many organelles, yet rich in hemoglobin (Hb) (270 million Hb molecules carried by each RBC), which is susceptible to auto-oxidation during circulation, leading to the loss of oxygen-supply capacity together with the production of toxic substances, such as ferryl-Hb. To avoid toxic substances generated by oxidative damage to hemoglobin during oxygen transportation, RBCs possess a system consisting of special enzymes such as catalase 36, 37. Therefore, we reasoned that using RBC membranes to prepare a biomimetic nanocarrier could enhance the biocompatibility *in vivo* as well as catalyze H_2_O_2_ in tumor cells to alleviate hypoxia, thereby promoting the efficacy of SDT.

**Figure 1:**
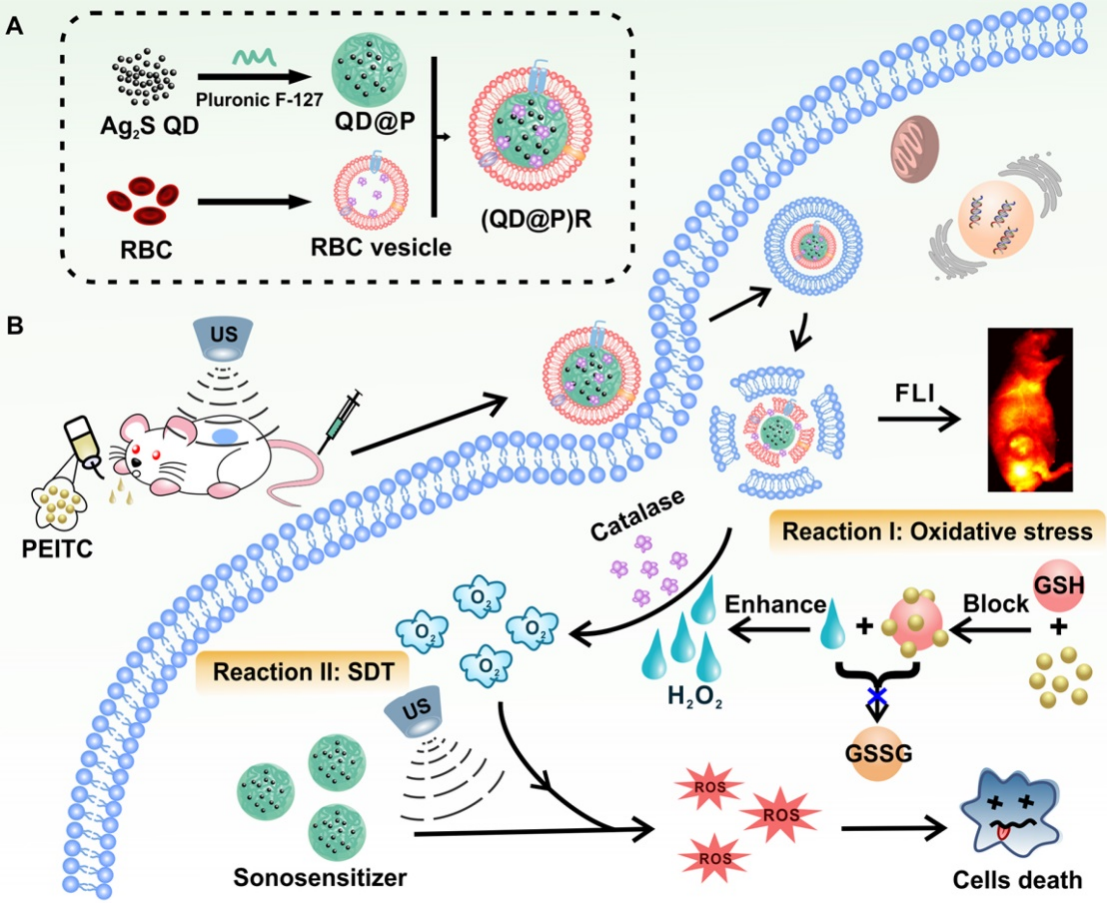
Schematic illustration of the synthesis route of (QD@P)Rs, tumor fluorescence imaging, and combined therapy.

A recent report showed that Ag_2_S QDs were a good fluorescence imaging agent* in vivo* and also had ideal photothermal and photodynamic therapeutic effects under laser irradiation, indicating that they could be an excellent multifunctional nanomaterial for tumor therapy 38. To date, application of Ag_2_S QDs as a sonosensitizer has not been investigated. In this study, we successfully constructed biomimetic nanoparticles (QD@P)Rs by wrapping Pluronic F-127-modified Ag_2_S QDs in RBC vesicles for enzyme-augmented SDT (Figure 1). The nanoparticles were employed *in vivo* for fluorescence image-guided non-invasive treatment of tumors in which, for the first time, Ag_2_S QDs were used as a sonosensitizer to generate ROS under ultrasonic stimulation. Encapsulation of the nanoparticles in RBC membranes not only prolonged the circulation time of the probe but also catalyzed endogenous H_2_O_2_ by the catalase in RBCs to ameliorate tumor hypoxia. Besides, US could also promote tumor blood flow, relieve the hypoxic condition, and enhance the SDT effect of the probe. *In vivo* experiments demonstrated that with the assistance of oral anti-tumor drug phenethyl isothiocyanate (PEITC), biomimetic (QD@P)R nanoparticles could effectively eliminate tumors and extend the survival time of mice. This novel nanoplatform provides an elegant approach for improving tumor hypoxia for the treatment of deep-seated tumors.

## Results and Discussion

### Synthesis and characterization of (QD@P)Rs

**Figure 2:**
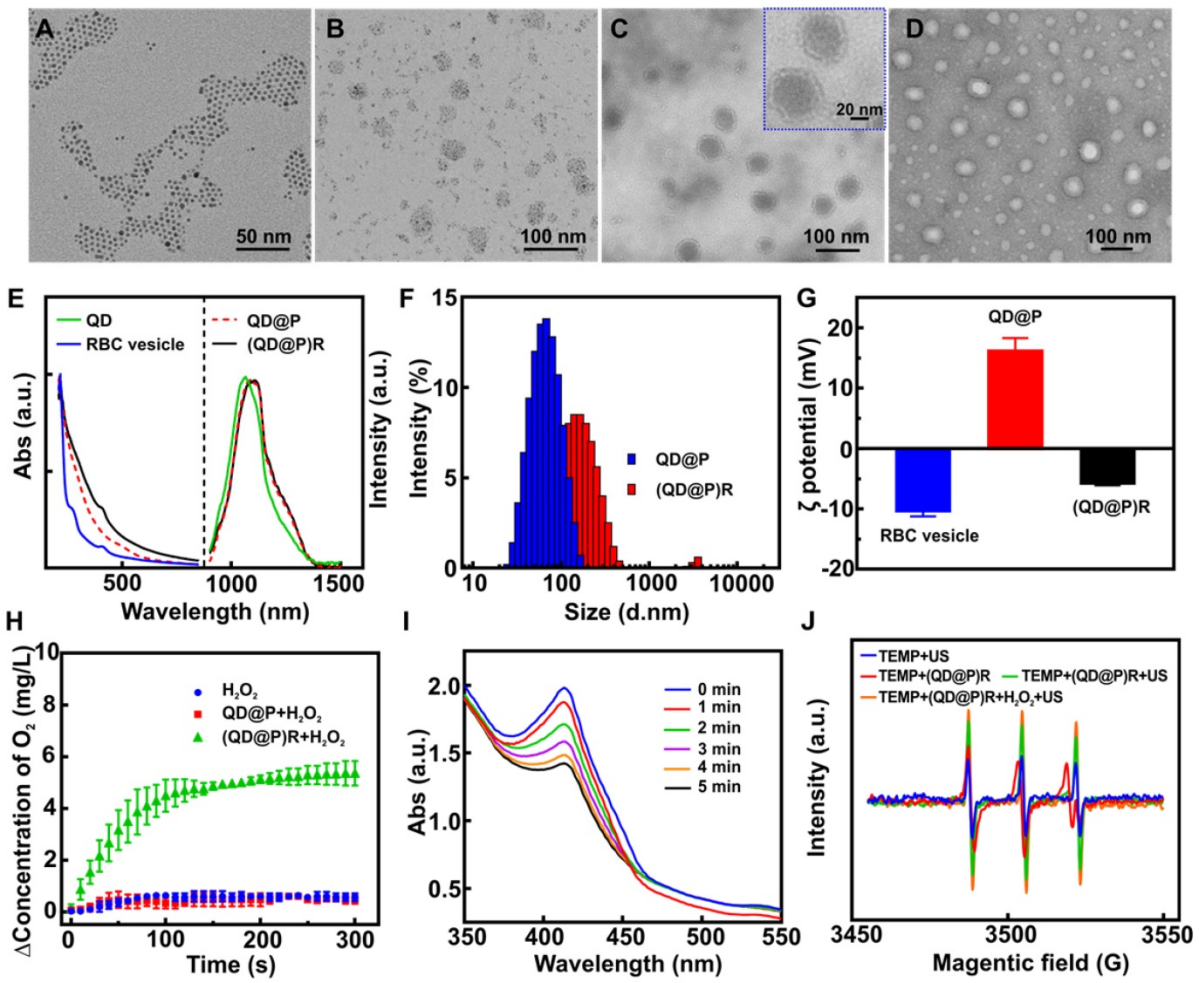
TEM images of Ag_2_S QD (A), QD@P (B), (QD@P)R (C), and RBC vesicles negatively stained with sodium phosphotungstate (D); UV-Vis absorption spectra, fluorescence spectra (E), hydrated particle sizes (F), and zeta potentials (G) of different nanoparticles; O_2_ generation by QD@P and (QD@P)R following addition of H_2_O_2_ (H); UV-Vis absorption spectra of DPBF in the presence of (QD@P)R upon US irradiation for prolonged duration (I); electron spin resonance (ESR) spectra of TEMP in the presence of (QD@P)R after different treatments (J).

Pluronic F-127, a triblock copolymer, has high biocompatibility *in vivo* and can effectively avoid protein adsorption, copolymer aggregation, and identification by the reticuloendothelial system, and has been approved by FDA for application as an intravenous drug 39. The PPO segment of Pluronic F-127 comprised of a hydrophobic core as a microenvironment for an oil-to-water phase transfer of Ag_2_S QDs, and the PEO segment of Pluronic F-127 prevented the adsorption and aggregation of the incorporated protein. Therefore, approximately 5 nm oil-soluble Ag_2_S QDs (Figure 2A) were uniformly encapsulated in the gel by using the hydrophobic structure of Pluronic F-127 to obtain hydrophilic QD@P nanomicelle of about 40 nm (Figure 2B). Subsequently, RBC vesicles (Figure 2D) of about 5-12 nm thickness obtained by the traditional hypotonic method were coated on the surface of QD@Ps through extrusion, and biomimetic composite nanoparticles, (QD@P)Rs, with uniform size of about 56 nm and good dispersion were obtained (Figure 2C). Fluorescence spectrum showed that the emission peak of oil-soluble Ag_2_S QDs was about 1050 nm under 808 nm excitation, and the peak emerged with a slight red shift after Pluronic F-127 wrapping and remained unchanged at 1100 nm after RBC vesicle coating (Figure 2E). UV-vis absorbance spectrum showed that (QD@P)R biomimetic nanoparticles presented characteristic absorption peaks of cell membrane proteins at 410 and 280 nm (Figure 2E). SDS-PAGE protein assay also supported that the membrane proteins of RBCs were well preserved on (QD@P)Rs (Figure S1) providing evidence that the RBC membranes had been successfully coated on the surface of QD@Ps. A comparison of the hydrated particle size of QD@Ps before and after the wrapping of RBC membranes indicated that the size increased from 68 nm to 141 nm, as measured by dynamic light scattering (Figure 2F), due to the strong hydrophilicity of membrane proteins. The zeta potential was also reduced from 16.1 mV to -5.8 mV after coating with the negatively charged RBC membranes (Figure 2G). To further evaluate the stability of the nanoparticles, (QD@P)Rs were stored at 24 and 37 ºC for 3 d in water, PBS, and RPMI 1640 (10 % FBS) medium. The results showed that particle size, polydispersity index (PDI) and zeta potential did not change significantly (Figure S2), indicating good stability of the probe.

### Catalytic O_2_ production and ROS generating efficacy of (QD@P)Rs upon US irradiation

To increase the O_2_ content in tumor cells and enhance the therapeutic effect of SDT, the ability of biomimetic (QD@P)R nanoparticles to catalyze H_2_O_2_ and generate O_2_ was evaluated. The results showed that the catalase activity of RBC vesicles and (QD@P)Rs was 799.7 and 611.9 U/mL, respectively. Although the catalytic activity of the enzyme decreased after encapsulation, the biomimetic nanoparticles still maintained the ability to catalyze H_2_O_2_. The quantification measurement revealed that the QD@Ps wrapped with RBC membranes could significantly catalyze H_2_O_2_ to generate O_2_ (Figure 2H). Also, the rate and yield of O_2_ production was concentration-dependent (Figure S3A). Considering the acidic environment of the tumor tissue, the effect of pH on the catalytic activity of the enzyme was further evaluated. The results demonstrated that the weak acidic environment had no effect on the catalytic efficiency of the biomimetic nanoparticles (Figure S3B). The catalytic efficiency of (QD@P)Rs did not show a significant downward trend even after four cycles of H_2_O_2_ addition (Figure S3C) proving that (QD@P)Rs coated with RBC vesicles maintained excellent catalytic activity and stability.

The mechanism of ROS generation during US treatment is not fully understood; however, sonoluminescence is believed to be a key phenomenon to generate ROS, and the sonosensitizer is activated from a ground state to an excited state by ultrasonic cavitation. It may directly react with the surrounding oxygen molecule, transforming a hydrogen atom to form a free radical. On returning to the ground state, such excited state sensitizer can also release energy to generate singlet oxygen (^1^O_2_) 40. Typically, TiO_2_ as a photosensitizer is activated by light for the generation of an electron (e^-^) and hole (h^+^) because of its intrinsic semiconductor property, which can also induce cancer cell death by this unique SDT procedure 41. As an excellent semiconductor, we speculated that Ag_2_S QDs might also be excited by light to generate electron and hole, which could also generate ROS such as ^1^O_2_, hydroxyl radical (•OH), and superoxide radical (O^2-^) upon US activation.

To evaluate the sonocatalytic effect of (QD@P)R nanoparticles, 1,3-diphenylisobenzofuran (DPBF) was employed as ^1^O_2_ probe to monitor the US-triggered ^1^O_2_ generation by (QD@P)Rs, after exposing the mixture of nanoparticles and DPBF to US irradiation. With the extension of ultrasound time, the characteristic absorption peak of DPBF at 410 nm decreased significantly in both groups with or without RBC membrane coating, indicating the generation of ^1^O_2_ (Figure 2I and S4). Also, the probes with the same molar concentration (1 mM) were incubated with DPBF for US treatment to compare the SDT efficiency of Ag_2_S QDs and TiO_2_ 41. The absorption values of DPBF and mixed solution at 410 nm after US treatment (1.5 W/cm^2^) were detected every 1 min (Figure S5). The calculated rate constants for ROS generation were 0.0007 s^-1^ for TiO_2_ and 0.0013 s^-1^ for Ag_2_S QDs. These results showed that the ROS generation efficiency of Ag_2_S QDs was higher than that of TiO_2_, which was suitable for SDT. We also tested 2,2,6,6-Tetramethylpiperidine (TEMP), which is a typical spin-trapping agent for ^1^O_2_ and yields 2,2,6,6-tetramethyl-1-piperidinyloxyl (TEMPO) free radical after trapping ^1^O_2_ and causes the ESR signal to split into a characteristic triple signal. Although all groups showed the characteristic signal of ^1^O_2_, higher signal intensity was achieved by (QD@P)Rs after ultrasonic stimulation (Figure 2J). The strongest signal appeared after the addition of H_2_O_2_, indicating that the enzyme-catalyzed O_2_ generation could provide an oxygen source for SDT-induced ROS production to achieve a better therapeutic effect.

### Endocytosis and cytotoxicity of (QD@P)Rs

**Figure 3:**
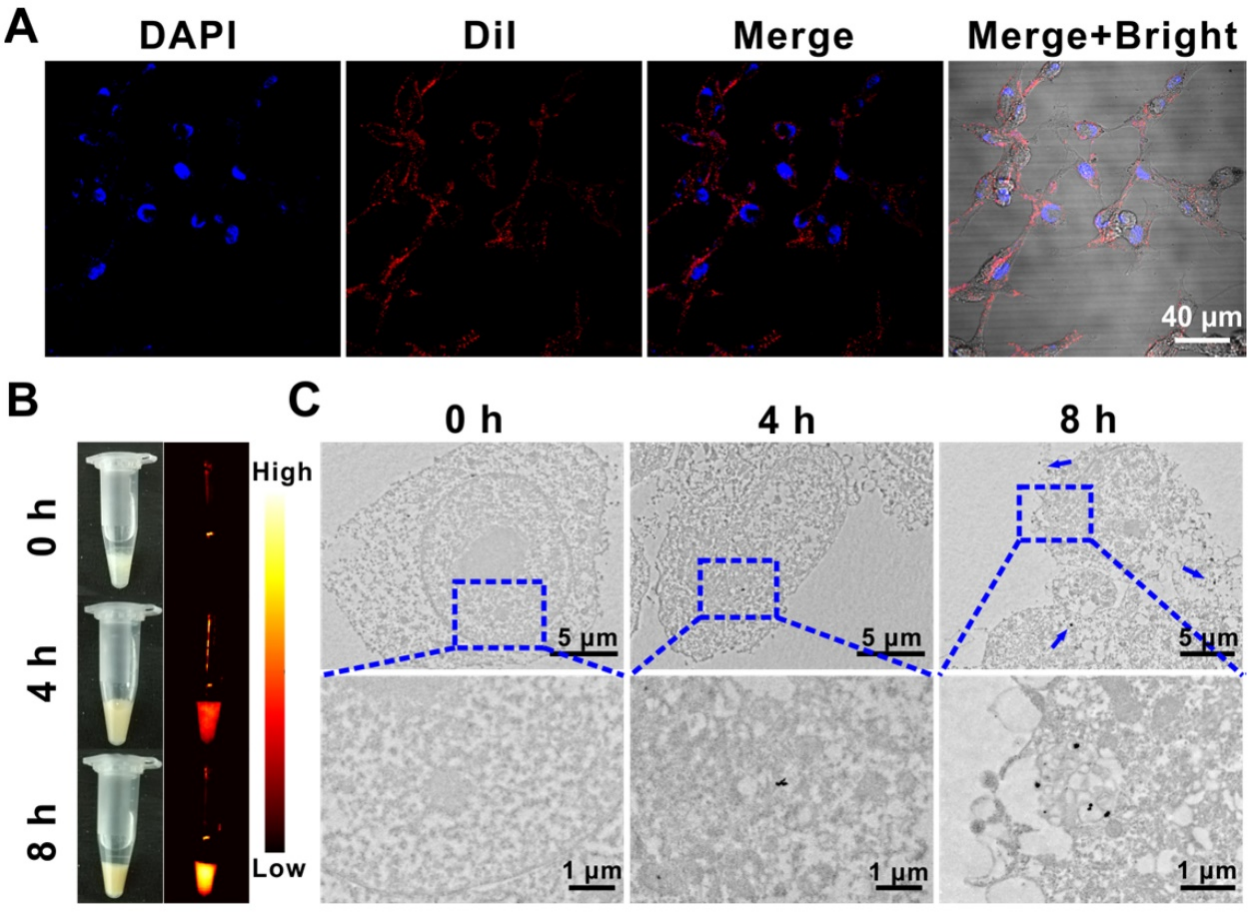
DiI-labeled (QD@P)Rs incubated with C26 cells for confocal fluorescence imaging (A); white light, NIR fluorescence (B), and biological TEM images of (QD@P)Rs incubated with C26 cell (C).

Compared with normal tissues, solid tumors have enhanced permeability and retention effect (EPR), which makes it easier for nanoparticles to selectively accumulate at the tumor site. Moreover, modification of the RBC membrane on the surface of nanoparticles could endow them with natural long circulation, low immunogenicity, and excellent biocompatibility 42, 43. Therefore, cell membrane fluorescent probe DiI was used to label the (QD@P)R biomimetic nanoparticles, which were then incubated with C26 cells to investigate the endocytosis of nanoparticles. Confocal images displayed that the labeled (QD@P)Rs entered into C26 cells after incubation for 4 h, and were mainly distributed in the cytoplasm (Figure 3A). NIR fluorescence (Figure 3B) and TEM (Figure 3C) images revealed that the intake of nanoparticles gradually increased with the extension of incubation time, indicating that more probe would accumulate in tumor cells with extended *in vivo* circulation time enabling efficient SDT treatment.

**Figure 4:**
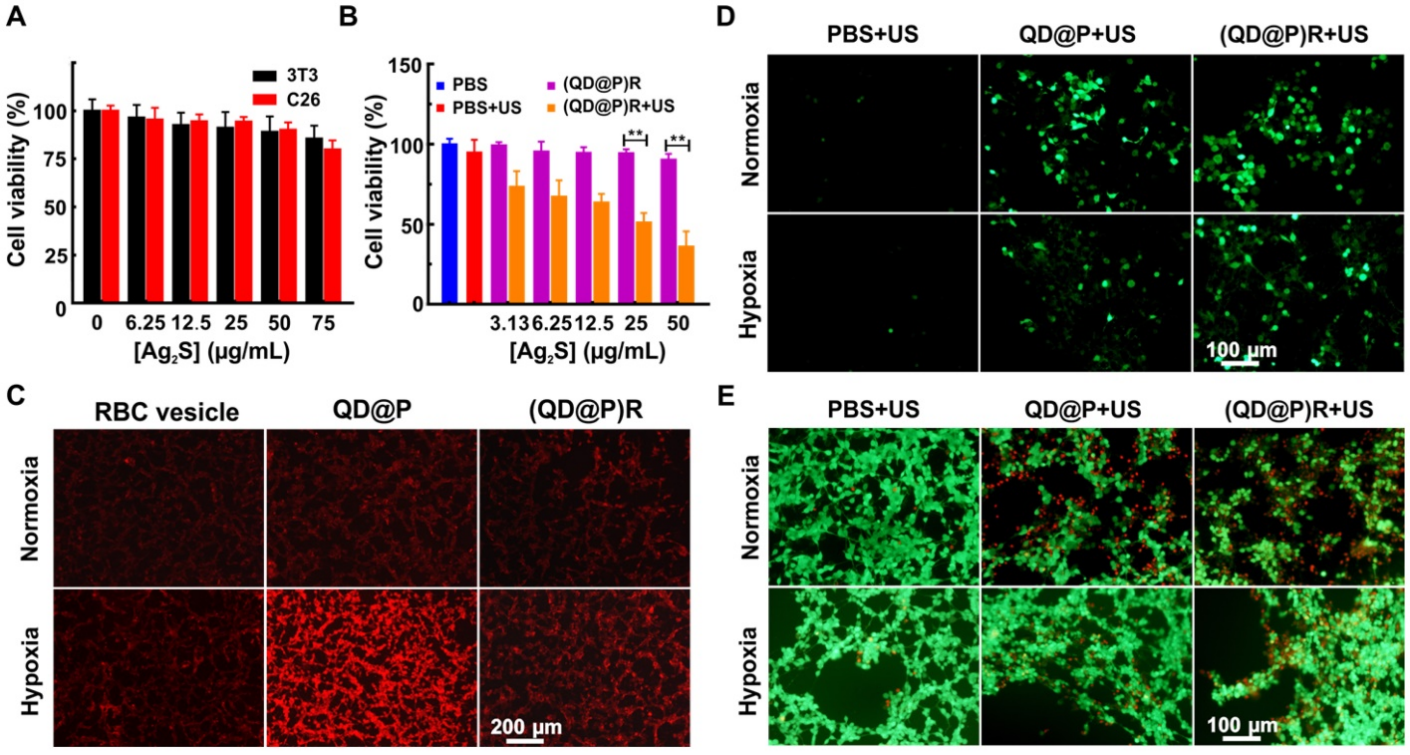
Cell viabilities of 3T3 and C26 cells incubated with (QD@P)Rs at different concentrations (A); cell viabilities of C26 cells treated with various probes after US irradiation (B); intracellular hypoxia imaging using [Ru(dpp)_3_]_2_Cl_2_ as the probe (C); intracellular ROS production detected by fluorescence of DCFH-DA (D); calcein and PI stained fluorescence imaging of C26 cells incubated with QD@Ps and (QD@P)Rs after US irradiation (E); **: *p*<0.01.

The safety of nanoparticles is of foremost importance for their potential bio-application. The cytotoxicity of (QD@P)Rs in 3T3 fibroblasts and C26 colon cancer cells were investigated by standard methylthiazolyl tetrazolium assay (MTT). The survival rates of normal 3T3 and C26 cells remained about 85.6±6.7 % and 80.1±4.6 %, respectively, after incubation with 75 μg/mL (QD@P)Rs (the probe concentration was represented by Ag_2_S QD concentration unless otherwise stated) for 24 h (Figure 4A). The viability of 3T3 cells and C26 cells treated with various concentrations of (QD@P)Rs was not significantly different, demonstrating low toxicity and good biocompatibility of the nanoparticles in both normal and cancer cells for the safety of subsequent animal experiments. Furthermore, the MTT assay was used to evaluate the SDT therapeutic effect of (QD@P)Rs on tumor cells (Figure 4B). The results showed that US irradiation had no significant effect on blank cells, whereas cells incubated with the probe showed therapeutic effect under US treatment even at a low concentration and cell death gradually increased with the increase in probe concentration. When the probe concentration was 50 μg/mL, the cell survival rate was only 36.1±9.6 %, proving that (QD@P)Rs had excellent SDT effect.

Next, the O_2_ production ability of (QD@P)Rs in C26 cell under hypoxia was investigated. To simulate the hypoxic environment of solid tumors, cells were pretreated with the hypoxia-mimetic agent deferoxamine, which was used as the fluorescent indicator of intracellular O_2_ and could strongly quench the red fluorescence of [Ru(dpp)_3_]Cl_2_. As shown in Figure 4C, only weak red fluorescence appeared in C26 cells for all three groups under normoxic condition. Interestingly, upon treatment with (QD@P)Rs under hypoxic condition, the intracellular fluorescence of [Ru(dpp)_3_]Cl_2_ was remarkably quenched compared with the non-membrane-modified QD@Ps, which were similar to that of RBC vesicle treatment group at the same concentration, indicating the high efficiency of (QD@P)Rs in elevating intracellular O_2_ content.

To study the intracellular mechanism of (QD@P)Rs as a sonosensitizer to kill cells, ROS generation under US irradiation was measured using DCFH-DA as a probe under normoxic and hypoxic conditions. Fluorescence images showed that under normoxic condition, US could not induce ROS generation without probe treatment, but both QD@Ps and (QD@P)Rs could greatly increase the intracellular fluorescence, implying efficient ROS generation (Figure 4D). However, when cells were exposed to hypoxia, ROS generation by QD@Ps was impaired, whereas (QD@P)Rs still displayed high ROS generation ability. Furthermore, calcein and propidium iodide staining was used to examine the SDT therapeutic effect of (QD@P)Rs on cells under normoxic and hypoxic conditions (Figure 4E). The results showed that under normoxic condition, the survival rates of cells incubated with QD@Ps and (QD@P)Rs were similar after irradiation by US. However, under hypoxia, the lethality of cells treated with (QD@P)Rs was significantly higher than that of QD@P-treated cells. Hence, these proved the advantages of (QD@P)Rs in cellular hypoxia modulation and enhanced SDT.

### *In vivo* biocompatibility and safety of (QD@P)Rs

Biocompatibility of a nanoprobe is an important factor for its application *in vivo*. Since the probe reaches the tumor site through blood circulation after injection, the hemolysis effect of the probe was examined. Only slight hemolysis was observed when (QD@P)R concentration was as high as 0.75 mg/mL (Figure S6), indicating that (QD@P)Rs had good blood compatibility and were suitable for intravenous administration enabling *in vivo* tumor treatment. Next, the effect of the probe on biochemical indexes of the mouse was investigated.

**Figure 5:**
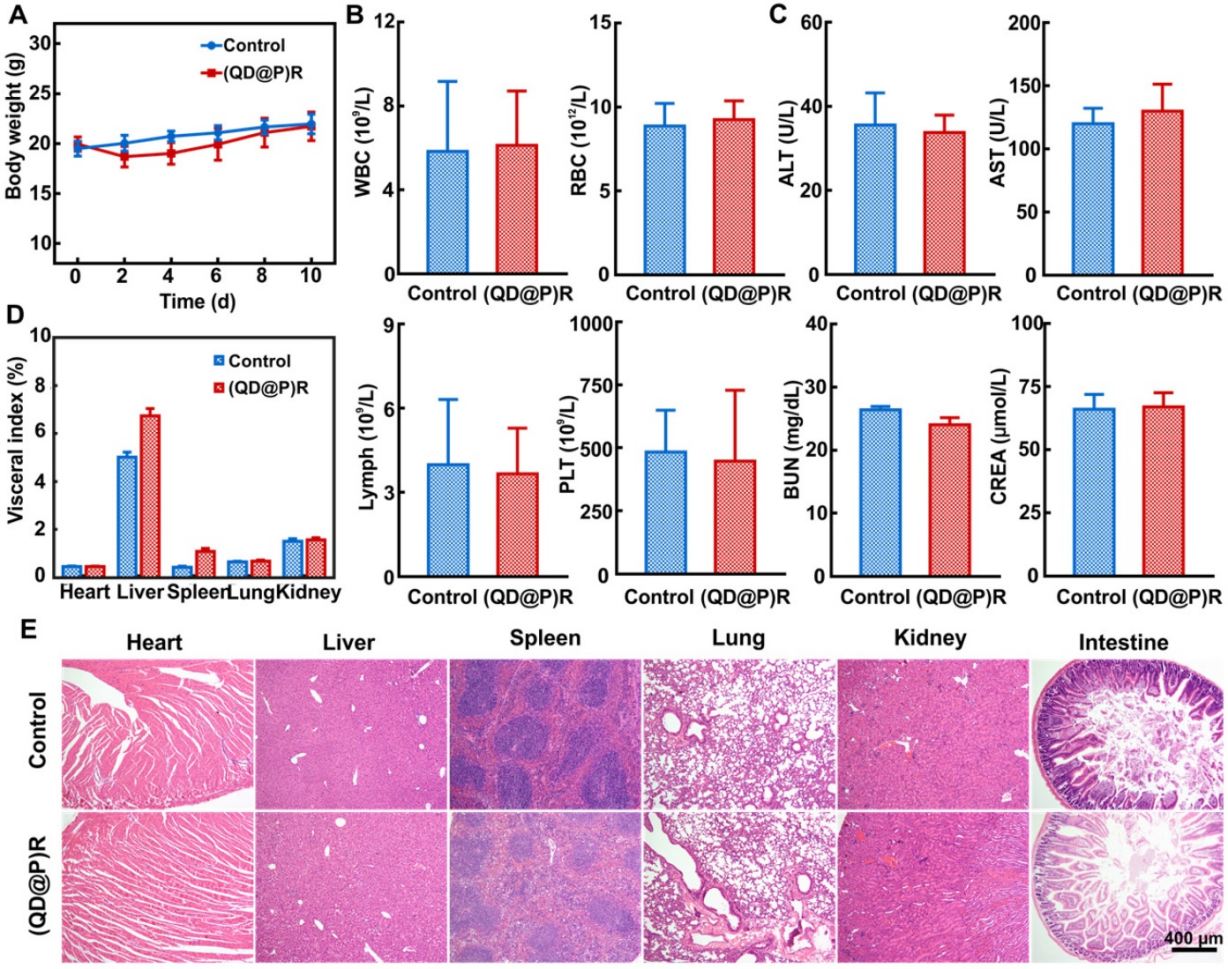
Changes in mice body weight (A); white blood cell count (WBC), red blood cell count (RBC), lymphocytes (Lymph), and platelets (PLT) (B); alanine aminotransferase (ALT), aspartate aminotransferase (AST), blood urea nitrogen(BUN), and creatinine (CREA) (C); various organ indexes (D) and H&E staining sections (E) within 10 d after injecting saline and (QD@P)R, respectively.

The results showed that the experimental group of tail vein injection of (QD@P)Rs was comparable to the control group of mice injected with saline in body weight within 10 d (Figure 5A). When the blood indexes of mice were measured, no significant difference was found in white blood cell (WBC), red blood cell (RBC), lymphocyte (Lymph) and platelet (PLT) counts between the experimental and control groups (Figure 5B). Also, there was no apparent difference in biochemical indexes of serum levels of alanine aminotransferase (ALT) and aspartate aminotransferase (AST), indicative of liver damage and blood urea nitrogen (BUN) and creatinine (CREA), parameters to determine renal function, of the (QD@P)R group when compared with the control group (Figure 5C). The organ indexes and H&E staining sections of main organs in both groups were also compared to determine whether the probe induced organ lesions. Statistical analysis showed (Figure 5D) no apparent difference in the indexes of heart, lung, and kidney between the experimental and control groups, but liver and spleen indexes increased slightly, indicating that there might be some organ influence. It is possible that as liver and spleen are the main metabolic and immune organs, the nanoparticles may be captured through the blood circulation and enriched, leading to the slightly higher values. However, compared with the control group, neither noticeable damage nor inflammation was observed from H&E staining images of liver and spleen in the experimental group (Figure 5E), and no significant pathological change was found in other organs. Therefore, our designed probe had good biocompatibility and could be further used for *in vivo* tumor treatment.

### *In vivo* NIR fluorescence imaging and enzyme-augmented SDT antitumor therapy

**Figure 6:**
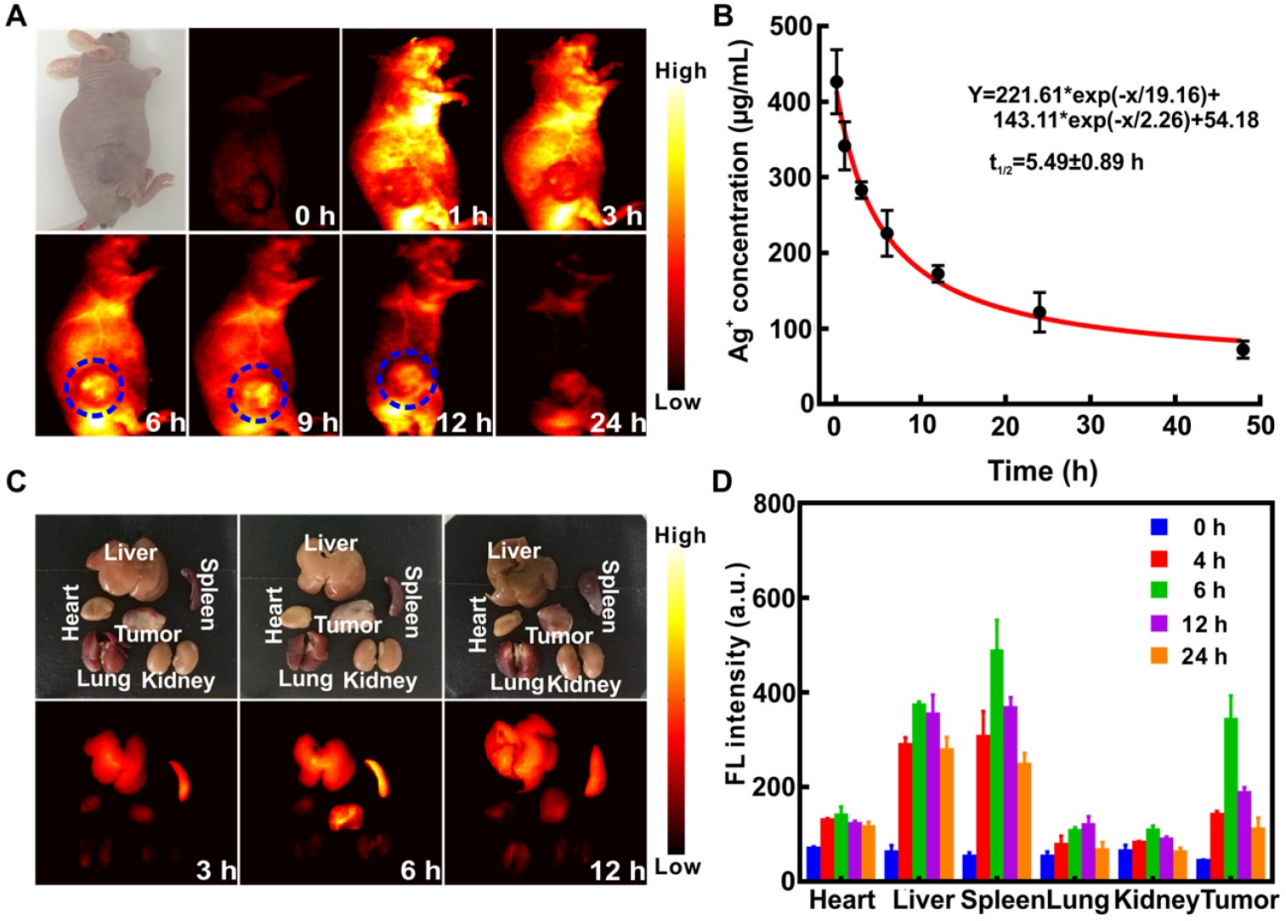
Fluorescence imaging of C26 tumor-bearing mice at different time points after intravenous injection of (QD@P)Rs (A); pharmacokinetics of (QD@P)Rs (B); white light and fluorescence imaging (C) and distribution (D) of organs and tumor at different time points after injection of (QD@P)Rs into C26 tumor-bearing mice.

Ag_2_S QDs possessed excellent NIR-II imaging ability, and the signal was visible in all organs 1 h after tail vein injection of (QD@P)Rs into C26 tumor-bearing nude mice (Figure 6A). The enrichment of probe at tumor site peaked at 6~9 h, and little fluorescence signal was observed after 24 h, indicating that the probe had been gradually excreted from the body. Pharmacokinetic results also showed that the circulation half-life of (QD@P)Rs was 5.49±0.89 h (Figure 6B), indicating that the coating of RBC membrane prolonged the probe blood circulation time and increased the enrichment of probe in the tumor. The fluorescence distribution results of major organs and the tumor also showed (Figure 6C and D) that the probe was first enriched in liver and spleen and gradually metabolized over time. The tumor fluorescence signal was strongest 6 h after the injection and then gradually weakened, which was consistent with the *in vivo* imaging results. Similar to the distribution results of the (QD@P)Rs group, QD@Ps were first enriched in liver and spleen (Figure S7). However, with time, the probe accumulated in the lungs which might be due to the partial degradation of the probe during blood circulation dispersing into small particles and enriching in the lungs. (QD@P)Rs were stable in blood circulation due to the RBC membrane coating. In summary, (QD@P)Rs constituted a satisfactory tumor imaging probe with a strong signal and long circulation time appropriate for guiding tumor SDT.

As an oral anti-tumor drug, PEITC has been shown to exert a noticeable chemo-preventive effect in a clinical trial 44. Among the various mechanisms of PEITC are apoptosis and cell-cycle arrest caused by oxidative stress. PEITC can also deplete glutathione (GSH) and inhibit glutathione peroxidase (GPX) enzyme activity to impair the glutathione antioxidant system. Due to vigorous metabolism of tumor cells with high GSH, PEITC causes preferential ROS (especially H_2_O_2_) accumulation in tumor cells compared with normal cells. Following oral administration of PEITC in C26 tumor-bearing mice for 2 d, the GSH and H_2_O_2_ content in tumor tissues were measured by kit-based procedures (Nanjing Jiancheng Bioengineering Co., Ltd.). The results showed that the post-treatment GSH content was 1.33±0.26 μmol/g lower than that in the untreated group (2.52±0.25 μmol/g), while the H_2_O_2_ content was increased to 1.15±0.17 mmol/g compared with 0.53±0.24 mmol/g in the untreated group. Therefore, PEITC was selected to synergistically enhance H_2_O_2_ content in the tumor cells along with generation of O_2_ catalyzed by the enzyme catalase of the (QD@P)R nanoparticles, thus providing ultrasound-triggered SDT to generate more ROS to inhibit tumor growth effectively.

**Figure 7:**
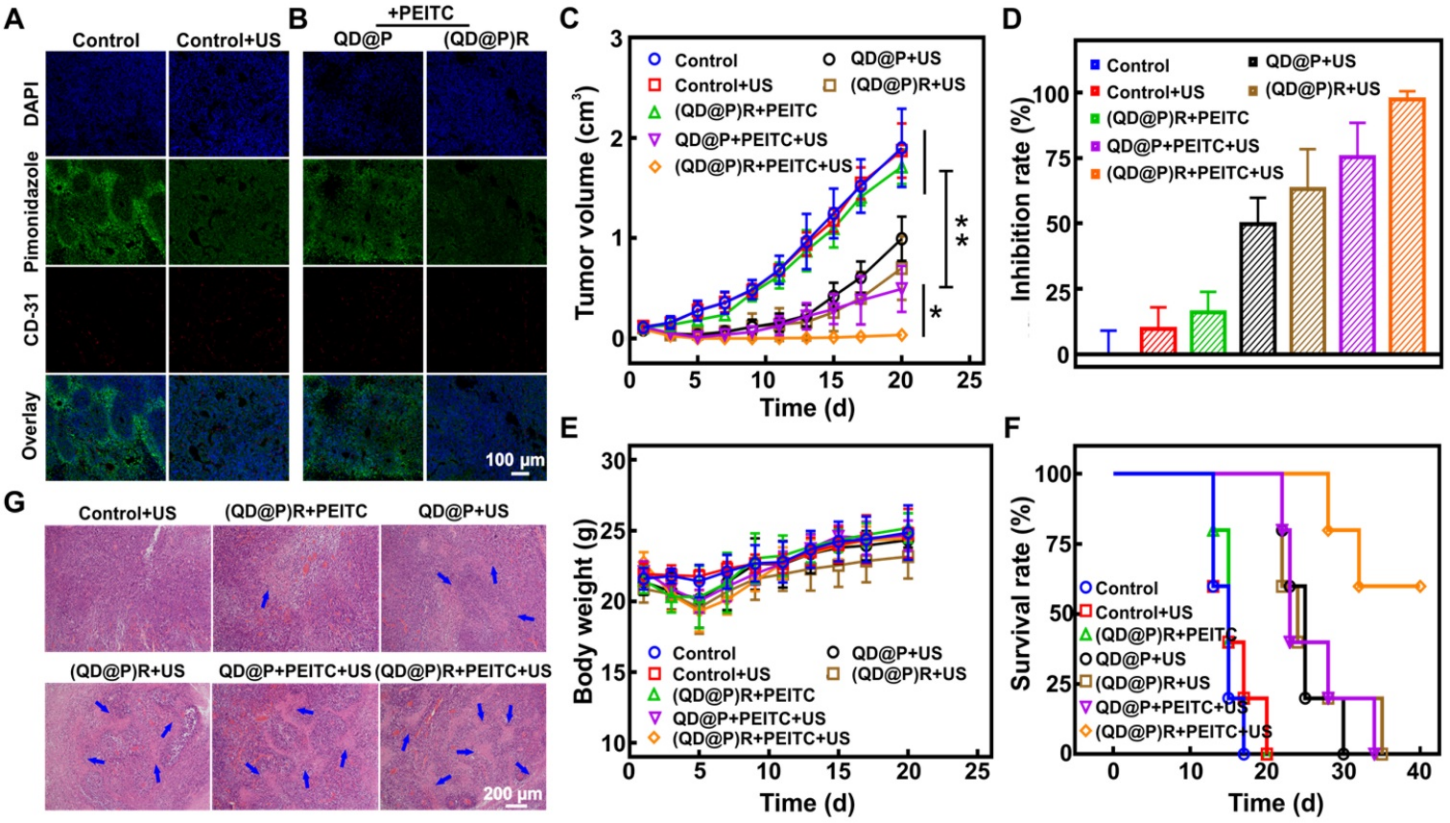
Immunofluorescence staining of O_2_ production in tumor site after US irradiation (A) and probe injection (B); tumor volume change (C), tumor inhibition rate (D), bodyweight change (E) and survival rate (F) of tumor-bearing mice with various treatments; H&E staining images of tumor after different treatments (G); *: *p*<0.05, **: *p*<0.01.

Immunohistochemical results showed (Figure 7A) that the fluorescence signal of the hypoxic probe Pimonidazole in C26 tumor tissues was significantly attenuated after US irradiation indicating that the fluorescence was quenched by O_2_ and that US stimulation could enhance the O_2_ content to alleviate tumor hypoxia. This might be because ultrasound could enhance local blood circulation and improve cell ischemia and hypoxia. C26 tumor-bearing mice were orally administrated with PEITC for 2 d, followed by intravenous injection of QD@Ps and (QD@P)Rs. The Pimonidazole fluorescence signal of the tumor site in the (QD@P)R group was considerably weakened compared with the QD@P group (Figure 7B). The signal intensity of the QD@P group was not significantly different from that of the control group without US irradiation, indicating that the (QD@P)Rs wrapped by RBC membrane could improve the O_2_ content of tumor cells by catalyzing H_2_O_2_.

Due to the limited single-treatment effect of SDT, the probe was intravenously injected twice, and the tumor site was also subjected to US irradiation twice at 6 and 9 h after each injection when the probe enrichment was highest. Mice were orally administrated with PEITC 2 d before treatment and were fed daily for a total of 5 d until the second treatment. The purpose of oral PEITC was to increase H_2_O_2_ at the tumor site during the US treatment, and it was conjectured that the reduction of administration dose would present little effect tumor treatment in our work 45. Analysis of the tumor volume (Figure 7C) showed a negligible effect of only US irradiation on the tumor, whereas, compared with controls, the US-triggered QD@P and (QD@P)R groups showed therapeutic effects during the initial treatment. However, the lack of O_2_ led to reduced efficacy of SDT. Although the RBC membrane-coated probe (QD@P)Rs could catalyze endogenous H_2_O_2_, there was no significant difference when compared with the untreated controls. The content of endogenous H_2_O_2_ in the tumor was higher than that in normal tissues, but it still needed to be further increased by oral PEITC. Also, the combination treatment of (QD@P)Rs and PEITC without US irradiation could not effectively inhibit tumor growth. However, the QD@P and (QD@P)R groups with oral administration of PEITC showed excellent therapeutic effects following US treatment (*p*<0.01) with 3 of the 5 mice injected with the (QD@P)R probe showing cell death and scarring after the first cycle of treatment (Figure S8). In the group of animals treated with QD@Ps without RBC membrane packaging, tumor recurrence was observed, while tumors in the (QD@P)R-treated group were significantly inhibited by augmented SDT, with almost no recurrence (*p*<0.05). Thus, the enhanced supply of O_2_ catalyzed by the enzyme catalase together with the assistance of PEITC promoted the production of ROS and significantly improved the anti-tumor efficiency (Figure 7D).

H&E staining of tumors from different treatment groups showed that the US-triggered (QD@P)R group generated the most ROS, and the tumor had the highest level of damage (Figure 7G). Furthermore, the body weight changes of mice in each group were consistent within 20 d (Figure 7E), indicating that the probe and treatment had no significant toxicity in mice. As per the ethical requirement, mice were sacrificed when the tumor volume exceeded 1500 mm^3^, and the survival curves of mice in each treatment group were further analyzed (Figure 7F). The results showed that all mice in the control group and the non-sonicated group died after 20 d of treatment and the mice injected with QD@Ps and treated with US began to die 22 d later and all died after 34 d. The (QD@P)R with PEITC group, however, maintained a 60 % survival rate up to 40 d after US stimulation, demonstrating that the treatment significantly prolonged the survival time of mice.

## Conclusions

In this study, we used, for the first time, Ag_2_S QDs as sonosensitizers to design and construct a multifunctional biomimetic nanoplatform (QD@P)R for SDT of the tumor. (QD@P)R nanoparticles, assisted by the anti-tumor drug PEITC, utilized catalase enzyme of RBC membrane to relieve tumor hypoxia, thereby further enhancing the SDT effect on the tumor under the guidance of fluorescence imaging. Ag_2_S QDs with fluorescence in NIR-II region were an excellent sonosensitizer, providing a novel strategy for the future design of a multifunctional theranostic nanoplatform.

## Experimental Section

### Synthesis of Pluronic F-127 encapsulated Ag_2_S QD (QD@P)

The method was modified according to the previously described procedure 46. 76.8 mg diethyldithiocarbamic acid silver salt (Ag (DDTC)), 30 g octadecene (ODE), and 6 g dodecanethiol (DT) were heated to 90 ºC with vigorous stirring under Ar for 10 min to remove water. The reaction was further heated to 150 ºC and maintained 10 min. In order to quickly reduce the temperature of the solution to prepare Ag_2_S QD, syringe was used to quickly add *n*-hexane to quench the reaction solution. Then triploid acetone was added and centrifuged at 12000 rpm for 10 minutes for cleaning twice. The hydrophobic Ag_2_S QD was re-dispersed in *n*-hexane, 100 mg Pluronic F-127 and 2 mg Ag_2_S QD were dissolved in 4 mL water and homogenized with ultrasound. The mixture was heated to 80 ºC, stirred rapidly to evaporate *n*-hexane completely, and the excess Pluronic F-127 was removed by dialysis.

### Reconstruction of red blood cell membrane on QD@Ps to synthesize (QD@P)Rs

Whole blood drawn from male Balb/c mice (6~8 weeks, purchased from Beijing Vital River Laboratory) was centrifuged at 3500 rpm for 5 min at 4 °C to remove the plasma and washed three times with ice-cold PBS to obtain RBCs. The washed RBCs were then lysed with 0.25×PBS in an ice bath for 2 h, centrifuged (8000 rpm, 10 min, 4 °C) and washed twice to collect RBC ghosts. The harvested RBC ghosts were sonicated in the ultrasonic bath at a frequency of 25 kHz and 100 W to prepare RBC vesicles. After ultrasonically mixing, 1 mL QD@P (C_QD_=2 mg/mL) was added to 1 mL RBC vesicle solution (1 mg/mL) and the membrane-coated (QD@P)Rs were prepared by extruding sequentially through 400 and 100 nm polycarbonate membranes with an Avanti mini extruder.

### *In vitro* detection of O_2_ generation

The generated O_2_ was detected by a dissolved oxygen meter. Before the measurement, (QD@P)Rs were dispersed in 15 mL deionized water, and the dissolved O_2_ was removed by bubbling Ar until it decreased to 0 mg/L. A variety of conditions were chosen for the experiment including different concentrations (10, 20, 30, and 40 μg/mL), pH values (pH=6.0, 7.4), and circulating H_2_O_2_. The final concentration of H_2_O_2_ used in the experiment was 1 mM.

### ^1^O_2_ production ability of (QD@P)Rs

By using the trapping agent TEMP, the ^1^O_2_ generation by US-activated (1 W/cm^2^, 2 min) (QD@P)Rs (100 µg/mL) was detected by an ESR (electron spin resonance) spectrometer. As controls, TEMP+US, TEMP+(QD@P)R, and TEMP+(QD@P)R+H_2_O_2_+US groups with or without US irradiation (1.0 MHz, 1.0 W/cm^2^, 2 min) were tested for comparison, and the final concentration of H_2_O_2_ was 1 mM. DPBF was used to analyze the production of ^1^O_2_ after ultrasonic treatment. The absorption value of the mixed solution of DPBF and (QD@P)R at 410 nm after US treatment (1.0 W/cm^2^) was detected every 1 min.

### Cytotoxicity and endocytosis

3T3 and C26 cell lines were cultured in DMEM and RPMI 1640 culture medium (Gbico, Invitrogen) containing 10 % FBS, respectively, and incubated in a humidified atmosphere containing 5 % CO_2_ at 37 °C.

Cytotoxicity was determined by the 3-(4,5-dimethylthiazol-2-yl)-2,5-diphenyl-2H-tetrazolium bromide (MTT) assay. The cells were seeded in 96-well plate (1×10^4^ cells per well) and incubated overnight, then treated with different concentrations of (QD@P)R for 24 h. Subsequently, 20 µL MTT (5 mg/mL) was added into each well and the cells were incubated for an additional 4 h. The medium was then discarded, 150 µL DMSO was added to solubilize formazan crystals, and a microplate reader was used to measure the absorption at 490 nm. Untreated cells were used as negative control.

C26 cells were seeded in 6-well plates (5×10^4^ cells per well) and grown for 24 h following which the medium was replaced with serum-free medium with 50 µg/mL (QD@P)Rs and the cells were cultured for another 4 and 8 h. Next, the cells were washed with PBS to remove the free probe, then immobilized with 2.5 % glutaraldehyde and collected for fluorescence and TEM imaging. For confocal imaging of cellular uptake of (QD@P)Rs, C26 cells were seeded in glass-bottom Petri dishes and incubated overnight. The cells were treated with DiI-labeled (QD@P)Rs for another 4 h, washed with PBS, and imaged by confocal microscopy.

### Detection of cellular O_2_

C26 cells were seeded in 24-well plates (1×10^4^ cells per well) and grown for 24 h. The cells were treated with RBC vesicles, QD@Ps and (QD@P)Rs (50 µg/mL). To induce the cellular hypoxia, deferoxamine (3×10^-5^ M) was added and liquid wax was used to form a liquid seal. After 4 h of incubation, 10 μL [Ru(dpp)_3_]Cl_2_ (final concentration: 30 μM) was added for 30 min. The cells were washed with PBS 3 times and imaged using an inverted fluorescence microscope.

### *In vitro* ROS generation

DCFH-DA was used as the ROS-monitoring agent. C26 cells were seeded in 24-well plates cultured overnight and treated with QD@P and (QD@P)R (50 µg/mL) for 4 h. The culture media was replaced with DCFH-DA (in RPMI 1640) for another 30 min staining. After washing with PBS, cells were irradiated with US (1 W/cm^2^) for 1 min and observed by fluorescence microscopy.

### *In vitro* antitumor therapy

To investigate the SDT property of the probe *in vitro*, C26 cells were seeded in 96-well plates, cultured overnight, and QD@Ps and (QD@P)Rs (50 µg/mL) were added. After for 4 h of incubation, PBS was used to remove the unbound probe. Subsequently, each well was irradiated by US (1 W/cm^2^, 1 min). The cells were stained with calcein and propidine iodide to observe the effect of SDT therapy.

The antitumor effect was also studied using the MTT assay. The C26 cells, seeded in 96-well plates and cultured overnight, were treated with different concentrations of (QD@P)R for 4 h. After washing with PBS to remove the unbound probe, cells were irradiated with US (1 W/cm^2^) for 1 min or without any treatment. The cells were then grown for another 24 h and cell viability was measured.

### *In vivo* toxicity evaluation of (QD@P)Rs

Male Balb/C mice were randomly divided into two groups (n=5), and intravenously injected with saline and (QD@P)Rs (25 mg/kg) on the first and third day. Mice were sacrificed after 10 days of observation, blood and major organs (heart, liver, spleen, lungs, kidneys, and small intestine) were collected, and weighed. H&E staining was performed to analyze the effect of the probe on mouse organs, and the visceral index was calculated as follows, organ mass/mice mass×100 %.

### *In vivo* NIR fluorescent imaging

4-5-week-old Balb/C nude mice were injected subcutaneously with 100 μL resuspended C26 cells (1×10^6^) to induce tumor formation. The treatment started when the tumor volume reached ~100 mm^3^ (0.5×length×width^2^). For *in vivo* imaging, (QD@P)Rs (25 mg/kg) were administered by tail vein injection in tumor-bearing mice and signal was acquired at different time points (0, 3, 6, 9, 12, and 24 h).

### *In vivo* hypoxia modulation

C26 tumor-bearing mice were treated with US at the tumor site for 5 min, or after tail vein injection of probes (QD@PR and (QD@P)R) for 9 h, the tumor was stripped for hypoxic and blood vessel (CD31) immunohistochemical staining. Pipenidazole was injected intraperitoneally 1.5 h before tumor resection (60 mg/kg).

### *In vivo* evaluation of SDT efficiency against tumor growth

C26 tumor-bearing mice with an average volume of ~80 mm^3^ were randomly divided into 5 groups and intravenously injected with different probes on day 1 and 3: (I) saline, (II) saline+US, (III) (QD@P)R+PEITC, (IV) QD@P+PEITC+US, (V) (QD@P)R+PEITC+US. The concentration of probes was 25 mg/kg. Groups II, IV, and V group were subjected to irradiation of tumor site with ultrasound (1.5 W/cm^2^) for 5 min after 6 and 9 h post-injection. Mice in III, IV and V groups received 5 µmol of PEITC in PBS by oral gavage daily from 2 d before probe injection and continued to day 3. At the end of treatment on day 3, the tumor was excised from one mouse from each group for H&E staining. Tumor volume and bodyweight of mice were measured every other day. As per ethical guidelines, mice were sacrificed when the tumor volume exceeded 1500 mm^3^, and the mice continued to be observed to analyze the survival rate. All animal experiments were approved by the Animal Experimental Ethics Committee of Huazhong University of Science and Technology.

## Supplementary Material

Supplementary figures.

## Abbreviations

SDT: sonodynamic therapy
ROS: reactive oxygen species
PDT: photodynamic therapy
RBC: red blood cell
PEITC: phenethyl isothiocyanate
PDI: polydispersity index
TEMPO: 2,2,6,6-tetramethyl-1-piperidinyloxyl
Ag (DDTC): diethyldithiocarbamic acid silver salt
ODE: octadecene
DT: dodecanethiol
ESR: electron spin resonance
MTT: 3-(4,5-dimethylthiazol-2-yl)-2,5-diphenyl-2H-tetrazolium bromide
DCFH-DA: 2,7-dichlorofluorescin diacetate
EPR: permeability and retention effect
DPBF: 1,3-diphenylisobenzofuran
TEMP: 2,2,6,6-Tetramethylpiperidine
