# Supplementary material for: Red blood cell membrane-enveloped O2 self-supplementing biomimetic nanoparticles for tumor imaging-guided enhanced sonodynamic therapy
Source: Theranostics. 2020 Jan 1;10(2):867–79. doi: 10.7150/thno.37930 (PMC6929970; doi:10.7150/thno.37930)
Supplement: Supplementary file 1 — Supplementary figures. [file thnov10p0867s1.pdf]

Supporting Information

Red blood cell membrane-enveloped O_2 self-supplementing biomimetic nanoparticles for tumor imaging-guided enhanced sonodynamic therapy

Cheng Li [a,b], Xiao-Quan Yang [a], Jie An [a], Kai Cheng [a], Xiao-Lin Hou [a], Xiao-Shuai Zhang [a], Yong-Guo Hu [a], Bo Liu [a], Yuan-Di Zhao [a,c,*]

[a] Britton Chance Center for Biomedical Photonics at Wuhan National Laboratory for Optoelectronics-Hubei Bioinformatics & Molecular Imaging Key Laboratory, Department of Biomedical Engineering, College of Life Science and Technology, Huazhong University of Science and Technology, Wuhan 430074, Hubei, P. R. China

[b] Beijing Advanced Innovation Center for Big Data-Based Precision Medicine, School of medicine, Beihang University, Beijing 100191, P. R. China

[c] Key Laboratory of Biomedical Photonics (HUST), Ministry of Education, Huazhong University of Science and Technology, Wuhan 430074, Hubei, P. R. China

* Corresponding author. Tel/Fax: +86-27-8779-2202. Email address: zydi@mail.hust.edu.cn (Y.D. Zhao).

Experimental Section

Materials

Diethyldithiocarbamic acid silver salt (Ag(DDTC), 98 %) and tris(4,7-diphenyl-1,10-phenanthroline)ruthenium(II) dichloride complex ($[Ru(dpp)_3]Cl_2$) were purchased from Aladdin Industrial Co., Ltd. (Shanghai, China); octadecene (ODE, 90 %), 1,3-diphenylisobenzofuran (DPBF, 97 %), phenethyl isothiocyanate (PEITC, 99 %), 2,7-dichlorofluorescin diacetate (DCFH-DA, 97 %) and Pluronic F-127 were purchased from Sigma-Aldrich (St. Louis, MO, USA); deferoxaminebmesylate was bought from Yuanye Technology Co., Ltd. (Shanghai, China). Oxygen-deficient marker Pimonidazole kit (Hypoxyprobe-1 Plus Kit) was purchased from Hypoxyprobe Inc. Other chemicals and reagents were used as received without any further purification.

Characterizations

Probe morphology characterization was performed by HT7700 (Hitachi, Japan) transmission electron microscopy. The absorption spectra were measured by UV-2550 UV-vis spectrophotometer (Shimadzu, Japan). The near infrared fluorescence (NIR) spectra of Ag_2S and QD@P were determined by NIRQUEST512-1.7 fiber spectrometer (Ocean Optics, USA). ZS90 ZetaSizer (Malvern, UK) was employed to characterize the size and surface potential of probe. The equipment also used in the experiment includes EMXmicro-6/1 ESR (Bruker, Germany), JPBJ-609L portable dissolved oxygen meter, ELX808IU microplate reader (Biotek, USA), FluoView FV1000 confocal fluorescence microscope (Olympus, Japan) and IX71 inverted fluorescence microscope (Olympus, Japan). NIR imaging system [1] was built up by our laboratory.

Pharmacokinetics and distribution of (QD@P)Rs

(QD@P)Rs (25 mg/kg) were injected *i.v.* into male Kunming mice. At 5 min, 1, 3, 6, 12 and 24 h after injection, blood was collected from the mice and dissolved in nitric acid to obtain the total amount of Ag^+ by graphite furnace atomic absorption spectrometry. C26 tumor-bearing mice were injected with (QD@P)Rs (25 mg/kg) intravenously, and the fluorescence intensity of different organs was measured by fluorescence imaging system at different time points.

Hemolysis assay

The blood compatibility of (QD@P)Rs were evaluated by hemolysis assay. 2 mL fresh mice blood was diluted with PBS to 4 mL and centrifuged at 3500 rpm for 5 min to isolate red blood cells (RBCs). The RBCs were further washed for five times and finally diluted to 20 mL PBS. Different concentrations of (QD@P)R were incubated with RBCs at 37 ℃ for 4 h, water as positive control and PBS as negative control. The absorbance of supernatants from each group was measured using microplate reader at 550 nm. Hemolysis percentage=$(OD_{test}-OD_{negative\ control})/(OD_{positive\ control}-OD_{negative\ control})\times100$ %.

Statistical Analysis

All data were presented as mean ±SD unless otherwise stated. All experiments were performed at least in triplicate. The statistical significance was determined using two-tailed Student's test (*$p<0.05$, **$p<0.01$) unless otherwise stated.

Supplementary Figures

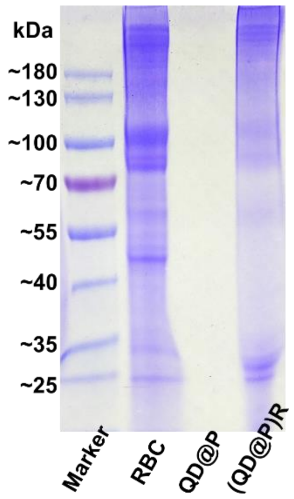

Figure S1. SDS-PAGE analysis of membrane protein changes.

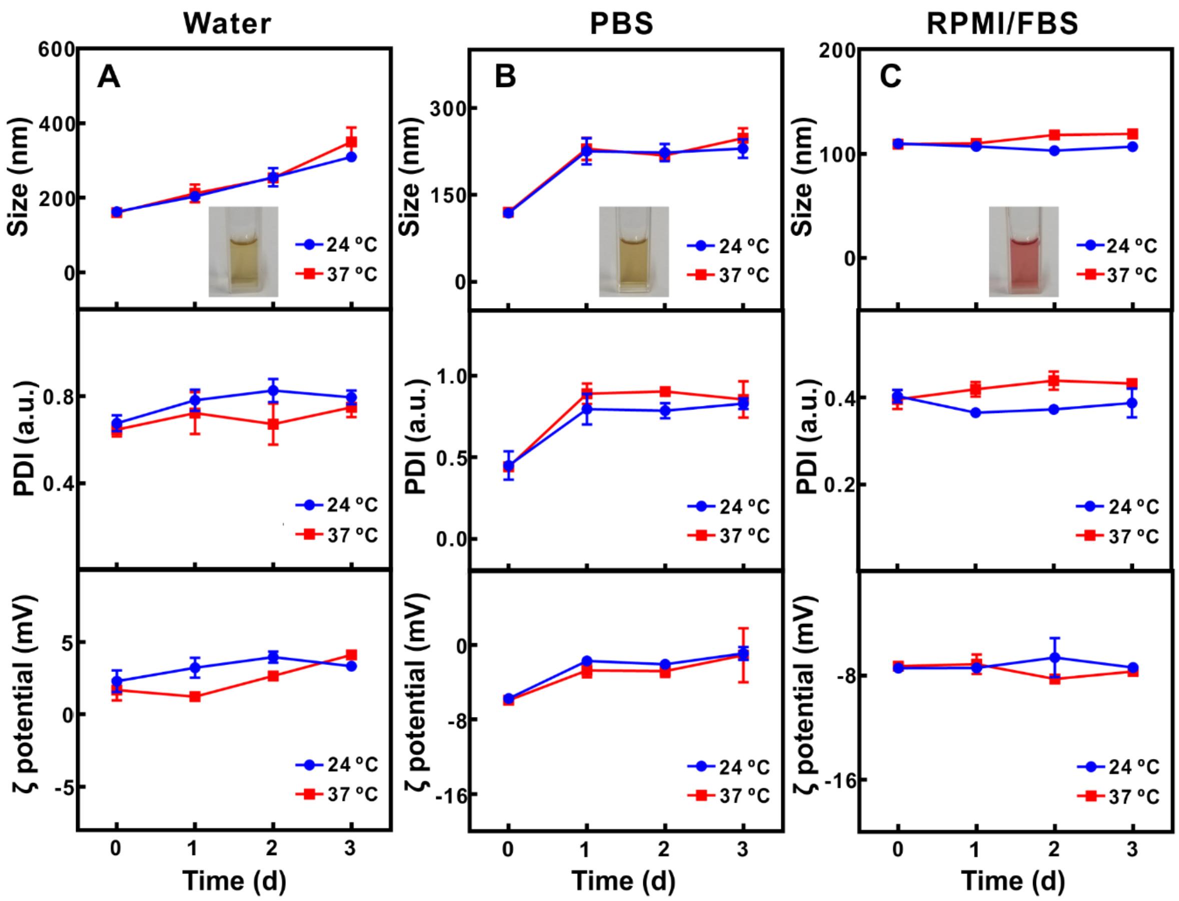

Figure S2. The change of hydrated particle size, polydispersity index (PDI) and zeta potential over time of (QD@P)Rs in water (A), PBS (B) and RPMI 1640 medium with 10 % FBS (C) at 24 and 37 ℃, respectively.

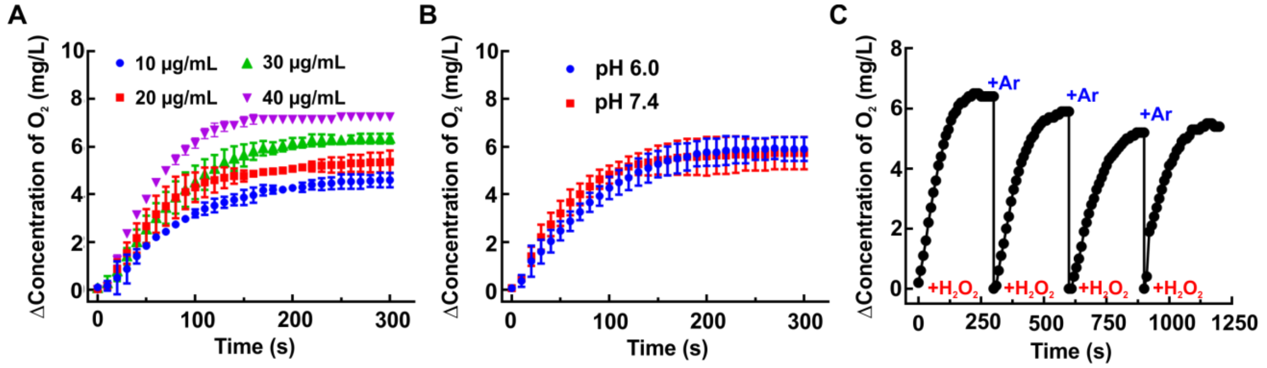

Figure S3. O_2 generation by tuning the concentrations of (QD@P)R (10~40 μg/mL) after incubating with H_2O_2 (1 mM) (A); O_2 generation by (QD@P)Rs (30 μg/mL) after incubating with H_2O_2 (1 mM) under different pH (B) and after cyclic additions of H_2O_2 (1 mM) (C).

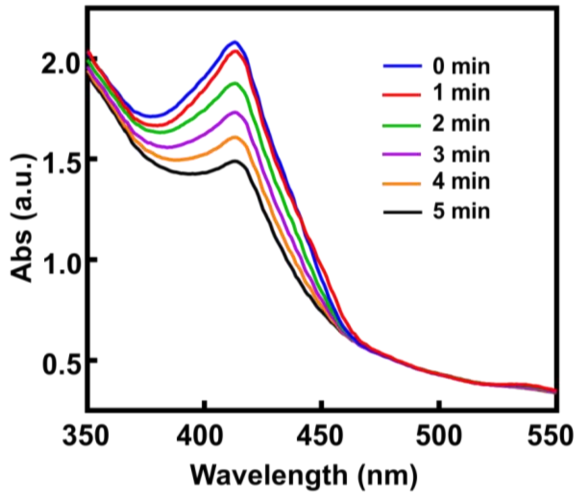

Figure S4. UV-Vis absorption spectra of DPBF in the presence of QD@Ps upon US irradiation for prolonged duration.

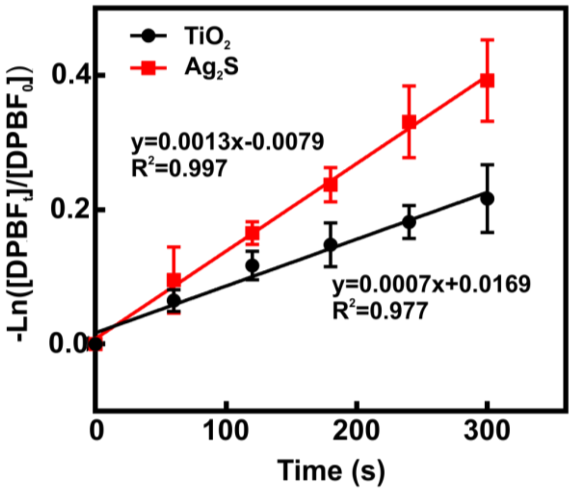

Figure S5. Firstorder plot of DPBF absorbance of TiO_2 and Ag_2S QDs versus time.

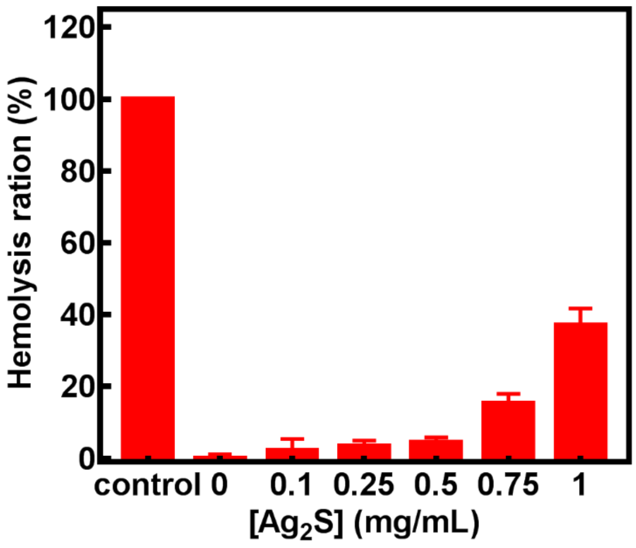

Figure S6. Hemolysis of (QD@P)Rs at various concentrations.

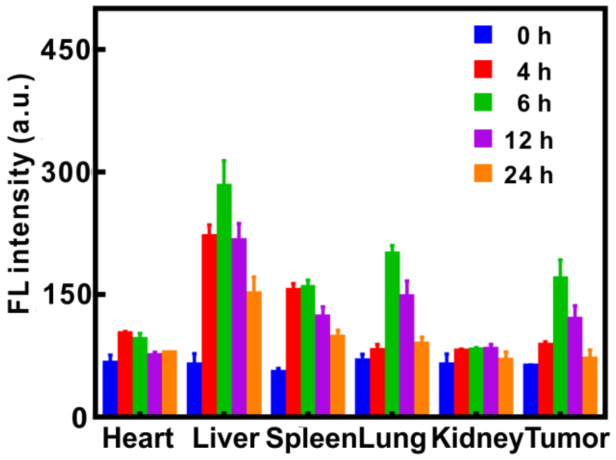

Figure S7. Distribution of organs and tumor at different time points after injection of QD@Ps into C26 tumor-bearing mice.

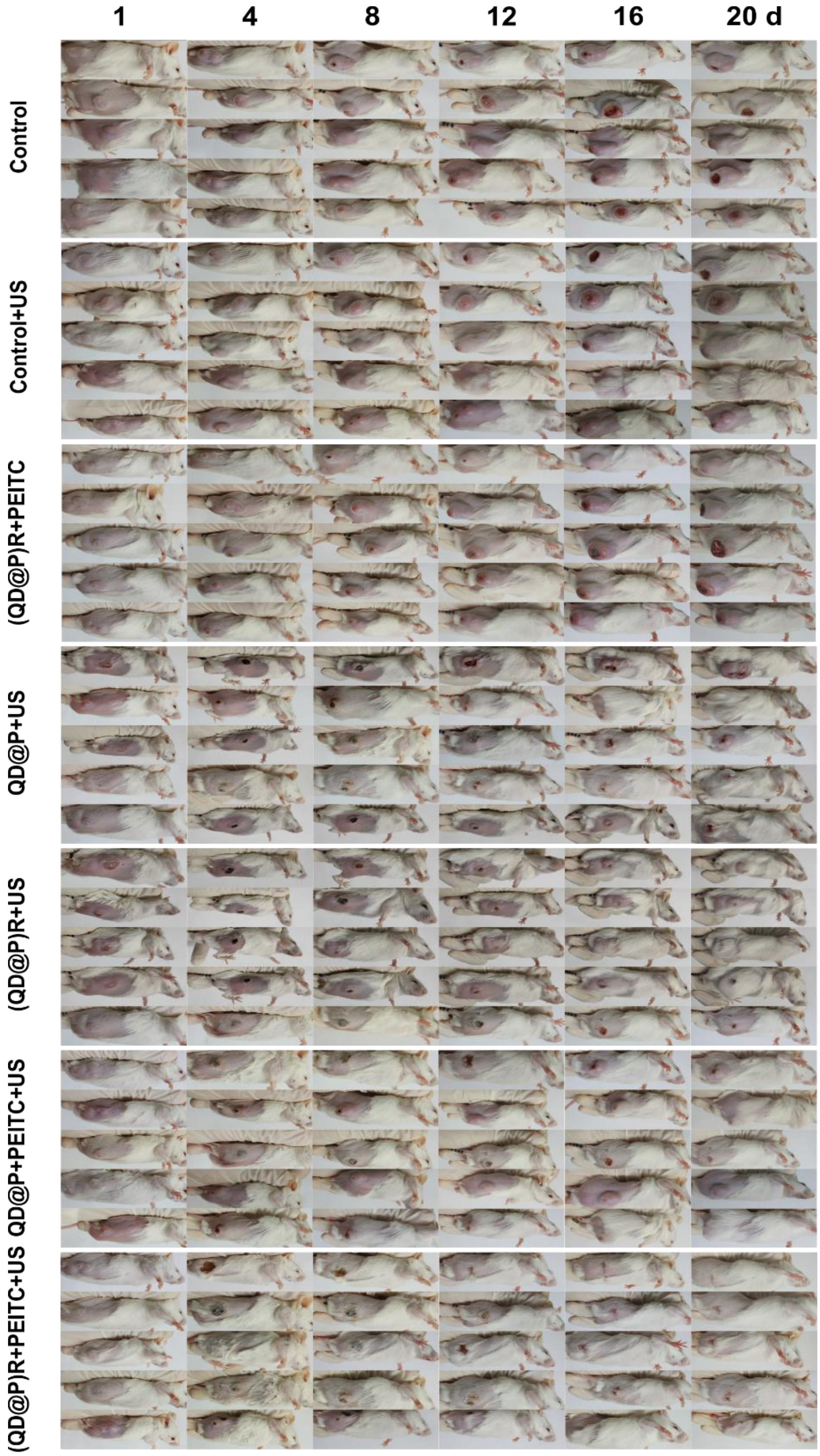

Figure S8. Images of mice with various treatments during 20 d.

References

[1] Wang K, Wang Q, Luo Q, Yang X. Fluorescence molecular tomography in the second near-infrared window. Opt Express. 2015; 23: 12669-79.
